# Immune Checkpoint Inhibitor-Induced Lymphocytic Esophagitis

**DOI:** 10.7759/cureus.39920

**Published:** 2023-06-03

**Authors:** Shefali Amin, Salina Munankami, Parth Desai, John Altomare, Nirav Shah

**Affiliations:** 1 Internal Medicine, Reading Hospital/Tower Health, West Reading, USA; 2 General Medicine, Kathmandu Medical College, Kathmandu, NPL; 3 Gastroenterology, Reading Hospital/Tower Health, West Reading, USA

**Keywords:** ici esophagitis, lymphocytic esophagitis, nivolumab, esophagitis, immune check-point inhibitor

## Abstract

Immune checkpoint inhibitors (ICIs) have emerged as effective treatments for a wide variety of advanced malignancies. However, their use is associated with numerous immune-related toxicities, including within the gastrointestinal tract. We present a rare case of checkpoint inhibitor-induced lymphocytic esophagitis. A 79-year-old male with a past medical history significant for metastatic renal clear cell carcinoma on nivolumab presented to the hospital with dysphagia and symptomatic choledocholithiasis. The patient underwent endoscopic retrograde cholangiopancreatography (ERCP) for the extraction of stones and esophagogastroduodenoscopy (EGD) for dysphagia, which showed esophagitis. Biopsies revealed lymphocytic infiltration of the epithelium, dyskeratotic keratinocytes, and acanthosis, raising suspicion for nivolumab-associated lymphocytic esophagitis. Treatment includes proton pump inhibitors and steroids; however, efficacy is not well described due to the rarity of the condition.

## Introduction

Immune checkpoint inhibitors (ICIs) have emerged as effective treatments for a wide variety of advanced malignancies [1]. Many immune system cells, such as CD4+ and CD8+ T cells, B cells, and natural killer cells, can up-regulate the expression of the PD-1 (programmed cell death 1) inhibitory receptor in the setting of lymphocytic activation [2]. This occurs in healthy individuals to prevent autoimmunity but can also occur in malignancies to escape immune surveillance [2]. Nivolumab is an ICI therapy that is a fully human IgG4 monoclonal antibody against PD-1 and has found success in treating many malignancies, such as advanced melanoma, renal cell carcinoma, and Hodgkin's lymphoma, among others [1,2]. However, its use is associated with numerous immune-related toxicities, notably colitis and hepatitis within the gastrointestinal tract, but rarely isolated esophagitis [2,3]. We present a rare case of immune checkpoint-inhibitor induced lymphocytic esophagitis.

## Case presentation

A 79-year-old male presented to the hospital with acute-onset right upper quadrant pain. His past medical history is significant for renal clear cell carcinoma status post-right nephrectomy with recurrent pulmonary and osseous metastases currently maintained on nivolumab maintenance therapy. The patient reported acute-onset severe abdominal pain shortly after eating a peanut butter sandwich. This pain was associated with nausea but without emesis. He had several similar intermittent episodes over the past few weeks, typically occurring after meals and spontaneously resolving within an hour. He also reported progressive dysphagia without odynophagia with solid foods and 15 pounds of weight loss over the past month due to fear of food getting stuck. He denied melena, hematochezia, change in bowel movement frequency or caliber, tobacco use, alcohol use, or a family history of any gastrointestinal cancer. Because this current episode persisted for more than an hour, the patient decided to present herself to the hospital for further evaluation.

**Figure 1:**
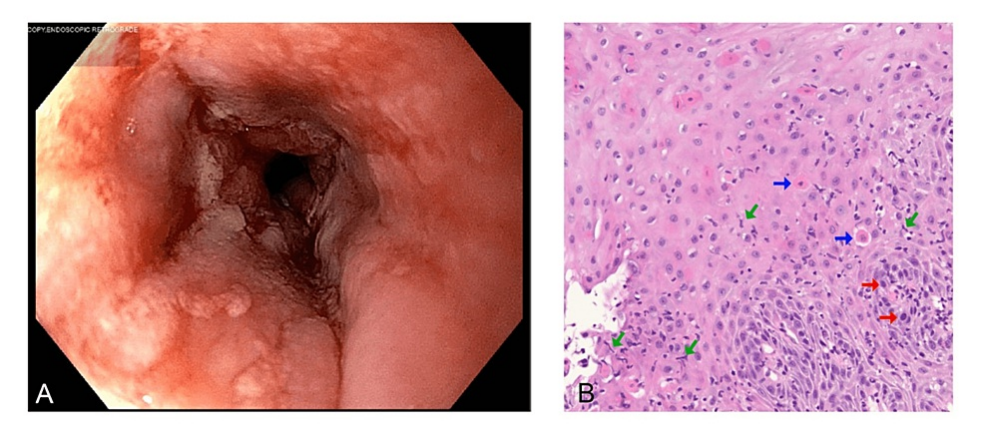
Endoscopy and pathology of ICI-induced esophagitis 1A. Endoscopy image showing an inflamed lower third of the esophagus with erythema and subtle furrows. 1B. Esophageal biopsy specimen demonstrating features of lymphocytic esophagitis. Inflamed squamous epithelium with an infiltrate of lymphocytes that appear as “squiggle cells” because of their stretched-out appearance (green arrows) and associated dyskeratotic keratinocytes (blue arrows). The red arrows indicate the basal keratinocytes.

In the emergency room, the patient arrived afebrile and hemodynamically stable. The physical exam was significant for scleral icterus, mild epigastric discomfort, and right upper quadrant tenderness with a negative Murphy’s sign. Initial labs were remarkable for acutely elevated hepatobiliary enzymes, as shown in Table 1. Right upper quadrant ultrasound revealed cholelithiasis, a dilated gallbladder, and a normal top common bile duct measuring 8 mm. Due to solid food dysphagia and signs and symptoms of obstructive jaundice with concern for choledocholithiasis, the patient underwent esophagogastroduodenoscopy (EGD) and endoscopic retrograde cholangiopancreatography (ERCP). ERCP was notable for the extraction of five pigmented stones from the common bile duct. EGD was notable for Schatzki’s ring at the gastro-esophageal junction, which was dilated to 15 mm, and an inflamed lower third and upper third esophagus with subtle furrows (Figure 1A). Biopsies were obtained. After the endoscopy, the patient was started on oral pantoprazole 40mg twice a day. After a successful inpatient laparoscopic cholecystectomy and symptomatic improvement, the patient was discharged home. Afterward, esophageal pathology revealed findings, including abundant lymphocytic infiltration of the epithelium, dyskeratotic keratinocytes, and acanthosis, raising suspicion for nivolumab-induced lymphocytic esophagitis (Figure 1B). The patient was started on a six-week prednisone taper, beginning with 40 mg, with plans for a repeat EGD two months after the index EGD.

**Table 1:** Admission lab values Admission lab values showing acutely elevated hepatobiliary enzymes.

	Admission Labs	Reference Range
Aspartate aminotransferase (AST)	250 IU/L	13-39 IU/L
Alanine transaminase (ALT)	331 IU/L	7-52 IU/L
Alkaline phosphatase	429 IU/L	34-104 IU/L
Total bilirubin	4.6 mg/dL	0.3-1.0 mg/dL
Direct bilirubin	3.2 mg/dL	0.0-0.3 mg/dL

## Discussion

Immune checkpoint inhibitors, such as nivolumab, a fully human IgG4 monoclonal antibody against PD-1, are novel therapies that have established significant efficacy and are increasingly used in various malignant conditions [1]. Of the many immune-related adverse events associated with these therapies, gastrointestinal toxicity is the most common type of toxicity, occurring in up to 35% of the population [2,3]. Although gastrointestinal complications can involve any segment of the gastrointestinal tract, they most commonly affect the colon [3]. ICI-related esophagitis, in particular, is exceptionally rare, with only a few cases reported in the literature describing esophageal toxicities, such as esophagitis and esophageal stenosis [3-5]. The median time from initiation of ICI use to the onset of esophagitis is approximately four months, in contrast to enterocolitis, which tends to have an onset of six to eight weeks [4,6]. The clinical presentation is comprehensive, with patients having low-grade esophagitis symptoms such as dysphagia, nausea, reflux, vomiting, and weight loss [2,3]. The diagnosis usually involves EGD with biopsy, which can demonstrate features ranging from mild inflammation to severe ulceration [3,7,8]. The pathophysiology is related to lymphocytic infiltration of the mucosa, which can also affect the muscular layer, causing ulcerations [9]. Given the lack of pathognomonic features of ICI-related esophagitis, a definitive diagnosis of ICI-related esophagitis cannot be made on histologic evaluation alone and remains a diagnosis of exclusion [3]. An important differential to consider is DRESS (drug reaction with eosinophilia and systemic symptoms) esophagitis. DRESS's esophagitis is also rare and can present with dysphagia; however, esophageal involvement occurs 3-4 weeks following exposure to the inciting medication, and EGD with biopsies on these patients has described significant mucosal eosinophilic infiltration [10]. Data for treating isolated esophagitis is based on the management of general adverse gastrointestinal reactions to checkpoint inhibitors [2]. One study from Panneerselvam et al. [3] showed success with proton pump inhibitors, while other studies from Liu et al. [11] have successfully treated the upper and lower gastrointestinal tracts with glucocorticoids. As the prevalence of ICI-related esophagitis is low, initial empirical therapy with proton pump inhibitors is recommended in the absence of alarming symptoms. If symptoms persist, endoscopy could be sought along with additional treatments, such as steroids with a slow taper [2]. In refractory cases, aggressive immunosuppressive therapy with infliximab may be considered, while ICIs should be permanently discontinued [2].

## Conclusions

Gastrointestinal toxicity manifests in up to 35% of patients treated with ICIs, with hepatitis and colitis being the most common pathologies. Isolated esophagitis is a rare adverse effect, and therefore, data on treatment is limited. Current recommendations for treatment include proton pump inhibitors and steroids; however, aggressive immunosuppressive therapy and discontinuation of ICIs may be warranted in severe cases. As the use of ICIs for the treatment of numerous malignancies increases, clinicians should be aware of the potential adverse event profile along with their knowledge of management and treatment.
